# A high temporal resolution numerical algorithm for shock wave velocity diagnosis

**DOI:** 10.1038/s41598-019-45112-3

**Published:** 2019-06-13

**Authors:** Yuji Wu, Feng Wang, Qiuping Wang, Yulong Li, Shaoen Jiang

**Affiliations:** 1 School of Physical Sciences, University of Science and Technology of China, Hefei, 230026 China; 2 Laser Fusion Research Center, Chinese Academy of Engineering Physics, Mianyang, 621900 China; 3 National Synchrotron Radiation Laboratory, University of Science and Technology of China, Hefei, 230026 China

**Keywords:** Nuclear fusion and fission, Applied mathematics, Optical sensors, Imaging and sensing

## Abstract

We propose a high temporal resolution numerical algorithm for shock wave velocity diagnosis. By analysing variations in the optical path and phasor of a light field, we determine a high temporal resolution shock wave velocity equation for a velocity interferometer system for any reflector (VISAR). The equation can be transformed into matrix form for numerical solution. To solve noise problems, a ‘filtering velocity spectrum’ method is proposed. Analysis of a VISAR data example shows that the resolution precision of shock wave velocity obtained from the numerical algorithm is the same as the temporal resolution of a streak camera. Moreover, it can observe the shock wave in greater detail. This algorithm can be used to observe detailed images and determine the mechanism and evolution of extreme shock waves, as well as provide data for research into hydrodynamic behaviour in inertial confinement fusion.

## Introduction

Shock wave propagation is often accompanied by a jump of material states, such as pressure, temperature, and density^1–3^. Furthermore, shock wave velocity can be a boundary condition for solving the equations of state that are widely used for high energy density physics research^4–8^. Particularly in inertial confinement fusion (ICF), the generation of shock waves in ablation is a high-speed passing process^9,10^. The entire process of propagation, reflection, attenuation, and disappearance only takes several nanoseconds or even picoseconds^11,12^. The driven asymmetry and fluid mechanics instability, which are the cause of compression insufficiency, can be reflected by the record of the shock wave^13–15^.

A velocity interferometer system for any reflector (VISAR) is considered a standard tool for shock wave information acquisition in ICF^16,17^. To meet the diagnostic needs of different conditions, the effects of imaging solid angle, window material, etalon, and noise fluctuation on VISAR calculation have been studied in recent decades^18–22^. It is generally considered that the temporal resolution of the VISAR technique depends on the temporal resolution of the conventional algorithm (equal to the etalon delay time, which is usually on the order of hundreds of picoseconds), the response time of the signal transmission system, and the temporal resolution of the recording system^23,24^. With the development of engineering, the latter two only affect the temporal resolution of the VISAR device by a few picoseconds; thus, the temporal resolution of the algorithm becomes the main factor that restricts the temporal resolution and improvement of VISAR detection technology. An improved shock wave velocity solution will enable the observation of transient physical processes, which may help researchers observe radiation matter interactions, as well as provide reliable experimental parameters for laser pulse shaping, converging geometry hydrodynamics, and astrophysics research^25–29^. As such, further improvements of temporal resolution are required to cope with rapid changes in shock wave velocity.

In this paper, we introduce a high temporal resolution numerical algorithm for VISAR. The algorithm consists of three parts. Firstly, a precise shock wave velocity and interference fringe phase equation is derived. Then, the equation is converted into a form that can be numerically operated. Finally, the noise amplification caused by numerical calculation is discussed and processed. Application of the numerical algorithm shows that the resolution precision of shock wave velocity is the same as the temporal resolution of a streak camera but can reveal the shock wave in greater detail.

## Derivation of the Phase Equation

**Figure 1:**
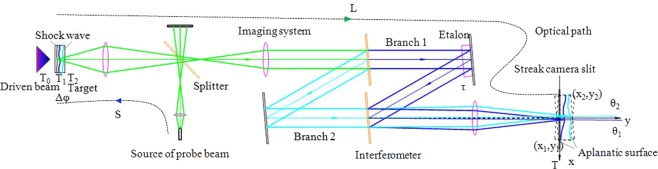
Simple VISAR schematic diagram for phasor analysis.

The principle of VISAR can be described by the simple schematic diagram shown in Fig. 1. The x-axis coincides with the slit of the streak camera and the y-axis coincides with the optical axis of VISAR. The etalon is set in branch 1. The solid angle for probe beam collection in VISAR discussed here is less than 7°; thus, the influence of frequency correction can be ignored^18^. First, the probe beam is incident on the shock wave interface through a beam splitter. Because of the optical Doppler effect, the reflected beam carries shock wave velocity information. The difference between the wavelength of the information beam and the probe beam is ${\rm{\Delta }}\lambda =-\,\frac{2u}{c}{\lambda }_{0}$, where *u* is the shock wave velocity, *c* is the speed of light, and *λ*_0_ is the wavelength of the probe beam. Then, the information beam enters the interferometer through the imaging system and is split into two beams by another splitter. One branch of the beams is delayed by the etalon with delay time $\tau =\frac{2h}{c}(n-\frac{1}{{n}_{0}})$, where *h* is the thickness of the etalon, *n* is the dynamic refractive index of the etalon, and *n*_0_ is the static refractive index of the etalon. Finally, the two beams interfere near the slit of the streak camera. By analysing the recorded phase shift of the interference fringe, the shock wave velocity can be obtained.

In the conventional algorithm, the number of dynamic waves in the etalon is considered to be $N=\frac{c\tau }{\lambda }$, where *λ* is the dynamic wavelength^22,30^. Thus, the phase shift of the interference fringe is

1 $$\begin{array}{rcl}\phi -{\phi }_{0} & = & 2\pi (N-{N}_{0})\\  & = & 2\pi c(\tfrac{\tau }{\lambda }-\tfrac{{\tau }_{0}}{{\lambda }_{0}})=2\pi c(\tfrac{{\tau }_{0}+{\rm{\Delta }}\tau }{{\lambda }_{0}+{\rm{\Delta }}\lambda }-\tfrac{{\tau }_{0}}{{\lambda }_{0}})\approx 2\pi c(\tfrac{-{\tau }_{0}}{{\lambda }_{0}^{2}}{\rm{\Delta }}\lambda +\tfrac{{\rm{\Delta }}\tau }{{\lambda }_{0}})\\  & = & 2\pi c(\tfrac{-{\tau }_{0}}{{\lambda }_{0}^{2}}{\rm{\Delta }}\lambda +\tfrac{\tfrac{2h}{c}\cdot \tfrac{dn}{d\lambda }\cdot {\rm{\Delta }}\lambda }{{\lambda }_{0}})=-2\pi c(\tfrac{-{\tau }_{0}}{{\lambda }_{0}^{2}}+\tfrac{\tfrac{2h}{c}\cdot \tfrac{dn}{d\lambda }}{{\lambda }_{0}})\cdot \tfrac{2u{\lambda }_{0}}{c},\\  & = & -2\pi (-\tfrac{{\tau }_{0}}{{\lambda }_{0}}+\tfrac{{n}_{0}{\tau }_{0}}{{n}_{0}^{2}-1}\cdot \tfrac{dn}{d\lambda })\cdot 2u=\tfrac{4\pi {\tau }_{0}u}{{\lambda }_{0}}(1-\tfrac{{n}_{0}{\lambda }_{0}}{{n}_{0}^{2}-1}\cdot \tfrac{dn}{d\lambda })\\  & = & \tfrac{4\pi {\tau }_{0}u}{{\lambda }_{0}}(1+\delta )\end{array}$$

where *N*_0_ is the number of static waves in the etalon, *φ* is the dynamic phase of the interference fringe, *φ*_0_ is the static phase of the interference fringe, and ${\tau }_{0}=\frac{2h}{c}({n}_{0}-\frac{1}{{n}_{0}})$ is the static delay time. The wavelength of the VISAR probe beam source at the SG-III prototype is 532 nm, and the etalon is fused silica. Therefore, *n*_*0*_ = 1.45847 and the correction factor is $\delta =-\,\frac{{n}_{0}{\lambda }_{0}}{{n}_{0}^{2}-1}\frac{dn}{d\lambda }=0.0318$^24,31,32^. After adding time coordinate *t* and position coordinate *x*, equation (1) can be rewritten as follows:

2 $$u(x,t)=-\,\frac{{\lambda }_{0}[\phi (x,t)-\phi (x,{t}_{0})]}{4\pi {\tau }_{0}(1+\delta )}.$$

Equation (2) expresses the relationship between the shock wave velocity and the fringe phase calculated by the conventional algorithm. Although *τ* and *λ* are considered variables, the conventional algorithm is established under the condition that the wavelengths of the probe beam in the etalon are equal everywhere. In addition, the optical path change between the target and the camera is not considered, and the optical path change of the target surface to the probe beam source is also neglected. Thus, the influence of the initial phase value of the two branches of the interference is not evaluated. These approximations reduce the temporal resolution of VISAR but can be considered by solving the phase vector equation. The following is a derivation of the relationship between the shock wave velocity and the fringe phase obtained using the phase vector equation.

Assuming the light field direction is perpendicular to the image plane, the phasor of the light field for branch *j* around the streak camera slit at *T* can be determined by the optical path:

3 $$\begin{array}{rcl}{U}_{j}(x,y,T) & = & {A}_{j}(x,y,T)\cdot \exp (i(\phi ({x}_{0},{y}_{0},{t}_{0})+{\rm{\Delta }}{\varphi }_{j}\\  &  & +\,2\pi (\frac{c}{{\lambda }_{0}}({T}_{j}-{t}_{0})-\frac{{S}_{j}}{{\lambda }_{0}}))),j=1\,or\,2,\end{array}$$

where ${A}_{j}(x,y,T)$ represents the optical amplitude of branch *j*, ${i}^{2}=-\,1$, $\phi ({x}_{0},{y}_{0},{t}_{0})$ is the initial phase of the light source, Δ${\varphi }_{j}$ is the phase shift of the information beam from the detection surface to the streak camera slit of branch *j*, *T*_*j*_ is the generation time for the information beam of branch *j*, and *S*_*j*_ is the optical path between the target and the streak camera at *T*_*j*_.

The following equations are the boundary conditions of equation (3):

4 $$\{\begin{array}{l}{S}_{j}={S}_{0}-{\int }_{{T}_{0}}^{{T}_{j}}\,udT\\ {T}_{1}+\frac{{L}_{1}}{c}={T}_{2}+\frac{{L}_{2}}{c}=T\\ {\rm{\Delta }}{\varphi }_{1}={\rm{\Delta }}{\varphi }_{1}\\ {L}_{j}={L}_{0}+(2-j)\tau c+(k-1)\,{\int }_{{T}_{0}}^{{T}_{j}}\,udT-\,\sin \,{\theta }_{j}(x-{x}_{j})+\,\cos \,{\theta }_{j}(y-{y}_{j}).\\ {x}_{j}={x}_{0j}-k\,\sin \,{\theta }_{j}\,{\int }_{{T}_{0}}^{{T}_{j}}\,udT\\ {y}_{j}={y}_{0j}+k\,\cos \,{\theta }_{j}\,{\int }_{{T}_{0}}^{{T}_{j}}\,udT\end{array}$$

where *T*_0_ is the static moment before compression, *S*_0_ is the optical path between the target and the streak camera at *T*_0_, *L*_*j*_ is the optical path between the light source and the target at *T*_*j*_, *L*_0_ is the optical path between the light source and the target at *T*_0_, *k* is the longitudinal magnification of the VISAR imaging system, *θ*_*j*_ is the angle between the light field propagation direction and the y-axis at branch *j*, (*x*_*j*_, *y*_*j*_) is the coordinate of the dynamic image, and (*x*_0*j*_, *y*_0*j*_) is the coordinate of the static image. In equation (4), the first term represents the optical path between the shock wave surface and the probe beam source; the second and third terms indicate that the probe beams reflected at different times are simultaneously recorded by the camera due to their passing through different branches; the fourth term represents the optical path between the streak camera slit and the shock wave; and the fifth and sixth terms represent the relationship between the coordinates of the static and dynamic images.

The light field at the slit of the streak camera is superimposed by two branches and the total light field intensity recorded by the camera is

5 $$I(x,y,T)={|{U}_{1}+{U}_{2}|}^{2}=({A}_{1}^{2}+{A}_{2}^{2})(1+\frac{2{A}_{1}{A}_{2}}{{A}_{1}^{2}+{A}_{2}^{2}}\,\cos \,2\pi (\frac{c}{{\lambda }_{0}}({T}_{1}-{T}_{2})+\frac{{S}_{2}-{S}_{1}}{{\lambda }_{0}})).$$

The experimental fringe pattern is generally filtered by the Fourier transform method; thus, the processed fringe pattern distribution is mainly determined by the phase change^33^. From equations (4) and (5), and the rotation transformation formulas

6 $$\{\begin{array}{l}{x}_{01}={x}_{0}\,\cos \,{\theta }_{1}-({y}_{0}-m)\,\sin \,{\theta }_{1}\\ {y}_{01}={x}_{0}\,\sin \,{\theta }_{1}-({y}_{0}-m)\,\cos \,{\theta }_{1}\\ {x}_{02}={x}_{0}\,\cos \,{\theta }_{2}-({y}_{0}-m)\,\sin \,{\theta }_{2}\\ {y}_{02}={x}_{0}\,\sin \,{\theta }_{2}-({y}_{0}-m)\,\cos \,{\theta }_{2}\end{array},$$

the following phase equation is obtained:

7 $$\begin{array}{rcl}\phi (x,T) & = & \frac{2\pi }{{\lambda }_{0}}(c({T}_{1}-{T}_{2})+{S}_{2}-{S}_{1})\\  & = & \frac{2\pi }{{\lambda }_{0}}(({\theta }_{1}-{\theta }_{2})(x+2({\theta }_{1}+{\theta }_{2})({y}_{0}-m))-\tau c-2\,{\int }_{{T}_{1}}^{{T}_{2}}\,udT).\end{array}$$

The purpose of the rotational transformation is to establish the relationship between the image coordinates in branches 1 and 2. Here, (*x*_0_, *y*_0_) is the static coordinate of the image plane when the light field direction coincides with the y-axis, and (0, *m*) is the intersection of the static images in branches 1 and 2 on the y-axis.

Equation (7) has three time-related moments: *T*, *T*_1_, and *T*_2_, whose relationship is described in equation (4). Thus,

8 $$\{\begin{array}{rcl}T & = & {T}_{1}+\tau +\frac{{L}_{0}}{c}+\frac{-\,{\int }_{{T}_{0}}^{{T}_{1}}\,udT-x\,\sin \,{\theta }_{1}+({y}_{0}-m)\,\cos \,2{\theta }_{1}}{c}\\ {T}_{2} & = & {T}_{1}+\tau +\frac{{\int }_{{T}_{1}}^{{T}_{2}}\,udT-({\theta }_{1}-{\theta }_{2})(x+2({\theta }_{1}+{\theta }_{2})({y}_{0}-m))}{c}\end{array}.$$

The change speed of the three time-related moments can then be compared,

9 $$\{\begin{array}{rcl}\frac{{\rm{\Delta }}T}{{\rm{\Delta }}{T}_{1}} & = & \frac{{\rm{\Delta }}{T}_{1}+{\rm{\Delta }}\tau -{u}_{{T}_{1}}{\rm{\Delta }}{T}_{1}/c}{{\rm{\Delta }}{T}_{1}} < 1+0.093\times {10}^{-3}+\frac{2\times 100}{3\times {10}^{5}}=1.00076\\ \frac{{\rm{\Delta }}{T}_{2}}{{\rm{\Delta }}{T}_{1}} & = & \frac{{\rm{\Delta }}{T}_{1}+{\rm{\Delta }}\tau -{u}_{{T}_{1}}{\rm{\Delta }}{T}_{1}/c}{{\rm{\Delta }}{T}_{1}(1-{u}_{{T}_{2}}/c)} < 1.00076/1.00067=1.00009\end{array}$$

Here, the change speed of T, T_1_, and T_2_ can be regarded as synchronous (non-uniformity is less than one thousandth). Then, considering the above conclusions and differentiating equation (7), we can obtain

10 $${\rm{\Delta }}\phi (x,t)=\frac{2\pi }{{\lambda }_{0}}\{-2\delta {\tau }_{0}{\rm{\Delta }}u(x,t)-2{\rm{\Delta }}t[u(x,t+{\tau }_{0})-u(x,t)]\}.$$

Integrating equation (10) and transforming it by Taylor expansion, we obtain

11 $$\begin{array}{rcl}-\frac{{\lambda }_{0}[\phi (x,t)-\phi (x,{t}_{0})]}{4\pi {\tau }_{0}(1+\delta )} & = & \bar{u}(x,t\to t+{\tau }_{0})-\frac{\delta [u(x,t+{\tau }_{0})-u(x,t)]}{1+\delta }\\  &  & -\,\frac{\delta {\tau }_{0}{\rm{\Theta }}u^{\prime} (x,t+{\rm{\Theta }}{\tau }_{0})}{1+\delta },\end{array}$$

where ${\rm{\Theta }}\in (0,1)$. Equation (11) reveals that the conventional equation between shock wave velocity and interference fringe phase $u(x,t)=-\,\frac{{\lambda }_{0}[\phi (x,t)-\phi (x,{t}_{0})]}{4\pi {\tau }_{0}(1+\delta )}$ is approximately a description of the average velocity in interval $(t,t+{\tau }_{0})$ (reflected in the first item of Taylor’s expansion), which explains why the temporal resolution of the conventional calculation cannot be less than *τ*_*0*_^24^. Moreover, the condition for establishing the conventional equation is that the change of *u* in interval $(t,t+{\tau }_{0})$ must not be overly large (reflected in the second item of Taylor’s expansion) or overly fast (reflected in the third item of Taylor’s expansion). The above conclusion is consistent with previous discussions of the application of the conventional equation to the numerical calculation of shock wave velocity^22,23,31,34^. When the shock wave velocity does not change rapidly, the conventional equation can easily calculate the velocity change caused by the phase change. However, when the shock wave velocity is generated, chased, or even oscillated, the instantaneous solution shown in equation (10) with a high temporal resolution provides a better description. Therefore, accurately solving equation (10) is crucial for shock wave velocity high resolution diagnosis.

## Numerical Calculation

Equation (10) describes the relationship between shock wave velocity and the VISAR fringe phase in the form of a differential equation. The VISAR recording based on the streak camera is an averaging process. Here, it is considered that each pixel records the average phase in the camera temporal resolution interval. Then, the solution of equation (10) cannot be less than the temporal resolution of the streak camera; thus, it is necessary to convert equation (10) into a form that can be used for numerical calculations. When $\bar{\phi }$ and $\bar{u}$ respectively represent the arithmetic average of the phase and velocity in the temporal resolution interval, equation (10) can be further changed to

12 $$\begin{array}{rcl}\bar{\phi }(x,t)-\bar{\phi }(x,t-\iota ) & = & \frac{2\pi }{{\lambda }_{0}}(-2\delta {\tau }_{0}({\bar{u}}_{t}-{\bar{u}}_{t-\iota })-2\iota ({\bar{u}}_{t+{\tau }_{0}}-{\bar{u}}_{t}))\\ \sum \,\{\bar{\phi }(x,t)-\bar{\phi }(x,t-\iota )\} & = & \sum \,\{\frac{2\pi }{{\lambda }_{0}}(-2\delta {\tau }_{0}({\bar{u}}_{t}-{\bar{u}}_{t-\iota })-2\iota ({\bar{u}}_{t+{\tau }_{0}}-{\bar{u}}_{t}))\}\\ \bar{\phi }(x,t)-\bar{\phi }(x,{t}_{0}) & = & \frac{4\pi \iota }{{\lambda }_{0}}(\begin{array}{c}-{\bar{u}}_{t+{\tau }_{0}}-{\bar{u}}_{t+{\tau }_{0}-\iota }\cdot \cdot \cdot -{\bar{u}}_{t+{\tau }_{0}-[\frac{{\tau }_{0}}{\iota }]\iota +\iota }-\frac{\delta {\tau }_{0}}{\iota }(\frac{{\tau }_{0}}{\iota }-[\frac{{\tau }_{0}}{\iota }]){\bar{u}}_{t+{\tau }_{0}-[\frac{{\tau }_{0}}{\iota }]\iota -\iota }\\ -(\frac{{\tau }_{0}}{\iota }-[\frac{{\tau }_{0}}{\iota }]+\frac{\delta {\tau }_{0}}{\iota }(1-\frac{{\tau }_{0}}{\iota }+[\frac{{\tau }_{0}}{\iota }])){\bar{u}}_{t+{\tau }_{0}-[\frac{{\tau }_{0}}{\iota }]\iota }\end{array})\end{array},$$

where *ι* is the temporal resolution of the streak camera. Equation (12) shows the average value of the shock wave velocity at each pixel of the camera and that the relationship between the fringe phase and velocity is nonlinear.

There are always slight data fluctuations superimposed with the true phase in the phase recording pattern that arise from the recording response, noise, phase extraction, etc.^31^. These fluctuations are random and difficult to completely eliminate. In particular, specific frequencies in the fluctuations may be amplified during the calculation. When the fluctuation is considered, equation (12) can be written as:

13 $${\bar{\phi }}_{\exp }(x,t)-{\bar{\phi }}_{\exp }(x,{t}_{0})-\bar{\omega }(x,t)=\frac{4\pi \iota }{{\lambda }_{0}}(\begin{array}{c}-{\bar{u}}_{t+{\tau }_{0}}-{\bar{u}}_{t+{\tau }_{0}-\iota }\cdot \cdot \cdot -{\bar{u}}_{t+{\tau }_{0}-[\frac{{\tau }_{0}}{\iota }]\iota +\iota }-\frac{\delta {\tau }_{0}}{\iota }(\frac{{\tau }_{0}}{\iota }-[\frac{{\tau }_{0}}{\iota }]){\bar{u}}_{t+{\tau }_{0}-[\frac{{\tau }_{0}}{\iota }]\iota -\iota }\\ -(\frac{{\tau }_{0}}{\iota }-[\frac{{\tau }_{0}}{\iota }]+\frac{\delta {\tau }_{0}}{\iota }(1-\frac{{\tau }_{0}}{\iota }+[\frac{{\tau }_{0}}{\iota }])){\bar{u}}_{t+{\tau }_{0}-[\frac{{\tau }_{0}}{\iota }]\iota }\end{array}),$$

where ${\bar{\phi }}_{\exp }(x,t)$ and $\bar{\omega }(x,t)$ are the recorded phase and the arithmetic average of the phase random fluctuation in interval *ι* at *t* respectively. If the velocity and phase values of each time interval are expressed in the above formula, it can be written in a matrix form,

14 $${\bf{A}}\cdot {\bf{u}}=-\,\frac{{\lambda }_{0}}{4\pi \iota }({{\boldsymbol{\Phi }}}_{\exp }-{{\boldsymbol{\Phi }}}_{\exp 0}-{\boldsymbol{\Omega }})$$

where

15 $${\bf{A}}=\{{A}_{dp}\}=\{\begin{array}{c}1,0\le d-p\le [\frac{{\tau }_{0}}{\iota }]-1\\ \frac{{\tau }_{0}}{\iota }-[\frac{{\tau }_{0}}{\iota }]+\frac{\delta {\tau }_{0}}{\iota }(1-\frac{{\tau }_{0}}{\iota }+[\frac{{\tau }_{0}}{\iota }]),d-p=[\frac{{\tau }_{0}}{\iota }]\\ \frac{\delta {\tau }_{0}}{\iota }(\frac{{\tau }_{0}}{\iota }-[\frac{{\tau }_{0}}{\iota }]),d-p=[\frac{{\tau }_{0}}{\iota }]+1\\ 0,d-p=else\end{array}\}.$$

Moreover, the shock wave velocity can be obtained from ${\bf{u}}=-\,\frac{{\lambda }_{0}}{4\pi \iota }{{\bf{A}}}^{-1}\cdot ({{\boldsymbol{\Phi }}}_{\exp }-{{\boldsymbol{\Phi }}}_{\exp 0}-{\boldsymbol{\Omega }})$. Here, **u** can be regarded as a one-dimensional matrix or as a velocity curve with respect to time, and the interval time of each element is the temporal resolution. Then, the shock wave velocity equation is transformed into a matrix describing the pixel points. It is then necessary to solve the shock wave velocity with a high temporal resolution (equal to the temporal resolution of the streak camera) through the matrix operation. Moreover, the key to solving the velocity is to remove the influence of noise from the operation process.

## Noise Filtering

We determine that **A** is similar to a Toeplitz matrix^35^; thus, **A**^−1^ is also similar to a Toeplitz matrix and the spectrum of discrete Fourier transforms for most rows of **A**^−1^ is approximately equal. Here, we term the discrete Fourier transform result of the velocity curve the ‘velocity spectrum’, thus each element of the velocity spectrum of **u** satisfies the following:

16 $$\begin{array}{rcl}\sum _{d=1}^{m}\,{u}_{d}{W}_{m}^{(d-1)(f-1)} & = & -\frac{{\lambda }_{0}}{4\pi \iota }(\sum _{d=1}^{m}\,\sum _{p=1}^{m}\,({A}_{dp}^{-1}({\phi }_{p}-{\phi }_{0}-{\omega }_{p}){W}_{m}^{(d-1)(f-1)}))\\  & = & -\,\frac{{\lambda }_{0}}{4\pi \iota }(\sum _{d=1}^{m}\,\sum _{p=1}^{m}\,({A}_{dp}^{-1}({\phi }_{p}-{\phi }_{0}){W}_{m}^{(d-1)(f-1)})\\  &  & -\,(\sum _{p=1}^{m}{\omega }_{p}{e}^{i\zeta (g)})|\sum _{d=1}^{m}{A}_{d\xi }^{-1}{W}_{m}^{(d-1)(f-1)}|)\end{array},$$

where *m* is the length of **u** and *d*, *f*, *p*, *ξ* are positive integers, $\xi \in (m/2,m)$.${W}_{m}={e}^{(-2\pi i)/m}$, $g=f+\frac{2\pi (p-\xi )}{m}$, $\zeta (g)$ is a function related to the noise phase and $|{e}^{i\zeta (g)}|\approx 1$. The second term on the right side of equation (16) is the source of amplified noise. It is difficult to calibrate noise in the experiment, but we can design a window such as $|\sum _{d=1}^{m}{A}_{d\xi }^{-1}{W}_{m}^{(d-1)(f-1)}|$ to filter it. Then, by performing inverse Fourier transform on the result, the real velocity curve can be obtained. Here, we present an example of the noise filtering.

**Figure 2:**
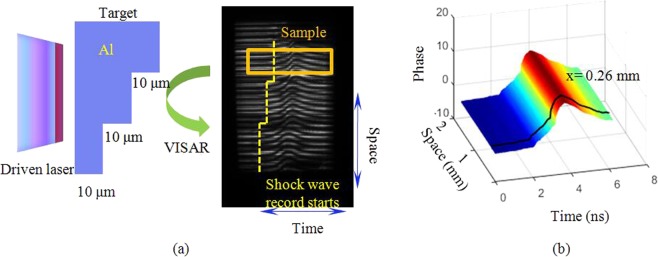
(**a**) Diagram of 20150522037 shooting data at the SG-III prototype facilityand (**b**) image of phase function **Φ**_exp_.

As shown in Fig. 2(a), we employ an experimental VISAR data set of 20150522037 shooting on the SG-III prototype facility. The target consists of three aluminium steps of different thicknesses. The probe beam reflected from the free surface is recorded by the VISAR. Because the driven laser pulse is modulated, the velocity rises with a certain gradient and gradually reduces owing to impact unloading. A part of the data set is selected for numerical calculation. It should be noted that the detection object of the VISAR can be a free surface or a shock wave surface, which is related to where the probe beam is reflected. The fringe pattern associated with the free surface velocity is chosen here because the data are representative and can be analogized to the shock wave velocity. An image of the phase function can be obtained through image processing (Fig. 2(b)). The temporal resolution of the streak camera is 20 ps. As the thickness of etalon is 17 mm, so the static delay time is ${\tau }_{0}=4.378\iota$.

**Figure 3:**
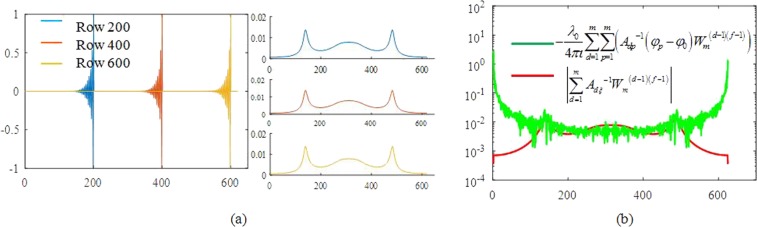
(**a**) Value of the 200^th^, 400^th^, and 600^th^ row of **A**^−1^ and their discrete Fourier transform spectra. (**b**) Value of $-\frac{{\lambda }_{0}}{4\pi \iota }\sum _{d=1}^{m}\,\sum _{p=1}^{m}\,({A}_{dp}^{-1}({\phi }_{p}-{\phi }_{0}){W}_{m}^{(d-1)(f-1)})$ when x = 0.26 mm and $|\sum _{d=1}^{m}\,{A}_{d\xi }^{-1}{W}_{m}^{(d-1)(f-1)}|$.

The discrete Fourier transform spectra of most **A**^−1^ rows are equal. Figure 3(a) shows the value of the 200^th^, 400^th^, and 600^th^ rows of **A**^−1^ and their discrete Fourier transform spectra. The value of $-\frac{{\lambda }_{0}}{4\pi \iota }\,\sum _{d=1}^{m}\,\sum _{p=1}^{m}\,({A}_{dp}^{-1}({\phi }_{p}-{\phi }_{0}){W}_{m}^{(d-1)(f-1)})$ when x = 0.26 mm and $|\sum _{d=1}^{m}\,{A}_{d\xi }^{-1}{W}_{m}^{(d-1)(f-1)}|$ is shown in Fig. 3(b). The coordinates of the peaks of the two curves coincide, which can be explained by equation (16). The second term on the right side of equation (16) is required to weight the function $|\sum _{d=1}^{m}\,{A}_{d\xi }^{-1}{W}_{m}^{(d-1)(f-1)}|$, so that noise at a specific frequency is enhanced in the calculation. Furthermore, we use $|\sum _{d=1}^{m}\,{A}_{d\xi }^{-1}{W}_{m}^{(d-1)(f-1)}|$ as a standard window to filter the first term and eliminate the effects of noise.

**Figure 4:**
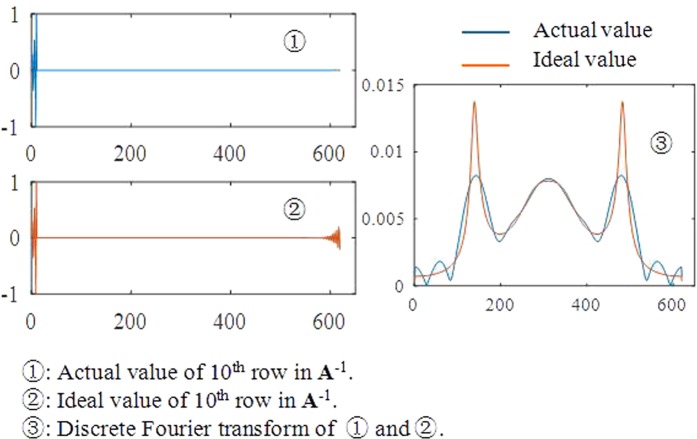
Actual and ideal value of the 10^th^ row and their Fourier transform values.

There are two aspects of the filtering process. One is that the Fourier transform spectra for the first few rows of **A**^−1^ are quite different from $|\sum _{d=1}^{m}\,{A}_{d\xi }^{-1}{W}_{m}^{(d-1)(f-1)}|$. For example, Fig. 4 is the value of the 10^th^ row in **A**^−1^ and the result of the Fourier transform. There are significant differences between the actual and ideal values in this case. This may cause a velocity turbulence near the initial time and the final time after filtering by window $|\sum _{d=1}^{m}\,{A}_{d\xi }^{-1}{W}_{m}^{(d-1)(f-1)}|$, which means that the new method sacrifices precision at the velocity curve boundary.

**Figure 5:**
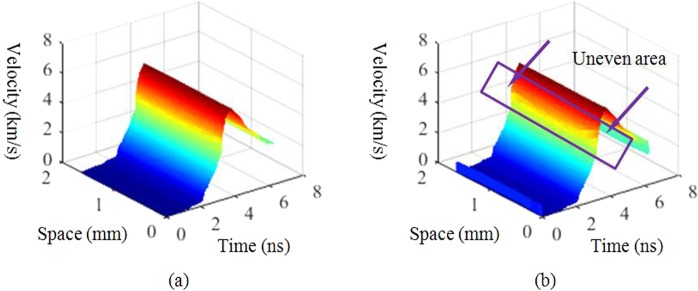
Diagram of the velocity field calculated by (**a**) the conventional algorithm and (**b**) the proposed algorithm.

The second aspect involves determining the weight of the window. The weight value is related to the random variable $\sum _{p=1}^{m}\,{\omega }_{p}{e}^{i\zeta (g)}$. One method estimates this value by observing the trend of $\sum _{p=1}^{m}\,{\omega }_{p}{e}^{i\zeta (g)}$ at the static fringe region. Another method adjusts the weight empirically by observing the possible relationship between the value of $-\frac{{\lambda }_{0}}{4\pi \iota }\sum _{d=1}^{m}\,\sum _{p=1}^{m}\,({A}_{dp}^{-1}({\phi }_{p}-{\phi }_{0}){W}_{m}^{(d-1)(f-1)})$ and the filter window. The choice of method depends on convenience for each case. In the VISAR data, the value of $\sum _{p=1}^{m}\,{\omega }_{p}{e}^{i\zeta (g)}$ is approximately 0.1 in the first 100 pixels and approximately 0.5 in the first 200 pixels; therefore, the overall value of $\sum _{p=1}^{m}\,{\omega }_{p}{e}^{i\zeta (g)}$ is 0.6–2.0. When $\sum _{p=1}^{m}\,{\omega }_{p}{e}^{i\zeta (g)}$ is taken as 1, a phase function is used as the input for both the conventional and proposed algorithms, and the velocity patterns of the entire space can be obtained as shown in Fig. 5.

A comparison of Fig. 5(a,b) shows that the velocity pattern obtained by the two algorithms are almost the same, except for the values in the rapid acceleration process. The proposed algorithm determines the uneven velocity distribution in impact loading, which indicates the possibility of uneven stress on the shock wave interface during acceleration. This uneven area only appears at approximately 400 ps then disappears. The temporal resolution of the conventional algorithm is approximately 100 ps; hence, this phenomenon is not clear. However, as the temporal resolution of the proposed algorithm is 20 ps, it can clearly identify this phenomenon.

It should be noted that the temporal resolution of the shock wave velocity obtained from proposed algorithm is the same as the temporal resolution of the recording device. In practice, the resolution of the streak camera is alterable owing to the parameter adjustment, and the etalon thickness is also variable because of experimental needs. When the etalon delay time is less or not significantly different from the temporal resolution of the streak camera, the difference between the two algorithm calculation results may be difficult to distinguish. However, because the spatial resolution of the camera is fixed, the speed resolution of the system decreases with the thickness of the etalon. Thus, the proposed algorithm has an advantage in terms of temporal resolution when a longer etalon is used and can provide a way to solve the shock wave velocity distribution when shock waves are generating, catching, or loading. On the other hand, the proposed algorithm is difficult to apply to the case where the fringe pattern has a jump. A few large non-random phase errors are amplified in the calculation and cannot be eliminated by existing filtering windows.

## Conclusion

To improve the temporal resolution of shock wave velocity in inertial confinement fusion, we proposed a numerical algorithm. Firstly, we reviewed the principle of VISAR as a standard piece of equipment for shock wave detection and noted that several conditions of the conventional algorithm reduce the temporal resolution. Then, by solving the phase vector equation of the light field near the detection surface, we obtained a precise differential equation for the shock wave velocity. To solve shock wave velocity, the equation was written in matrix form for numerical operations. We also determined that the noise fluctuation in the phase record is amplified during the calculation, so a filter window was designed for filtering the velocity spectrum. The shock wave velocity calculated by the algorithm has the same temporal resolution as the streak camera and can reflect velocity distribution information that is neglected by the conventional algorithm.

The proposed numerical algorithm provides a way to calculate shock wave generation, catching, and evolution at high temporal resolution, enabling precise observations of extreme fluid dynamic behaviour. In inertial confinement fusion, this algorithm could provide abundant data for researching laser-plasma interactions, hydromechanics instability, and driven laser tuning, as well as contribute to the creation of preparatory conditions for final ignition. Our numerical algorithm can even be extended as a calculation reference for other optical instruments containing an etalon, especially if there is a need for high temporal resolution.
